# Inaugural University of Queensland-Ochsner Doctor of Medicine Program, Year 3 Scholarly Project October 20, 2025

**DOI:** 10.31486/toj.26.5060

**Published:** 2026

**INTRODUCTION**

The curriculum for The University of Queensland (UQ) Doctor of Medicine (MD) program underwent a refresh in 2023 that touched each of the 4 years in the program. This refresh included the curriculum delivered at the UQ-Ochsner Clinical School (OCS) in New Orleans, Louisiana.

In academic year 2025, the third-year curriculum redesign impacted students in Brisbane, Australia, and in New Orleans. A year 3 scholarly project became a core element of the new MD program (MEDI7300), running concurrently with students’ clinical placements throughout the year.

At OCS, 18 faculty supervisors mentored groups of 4 to 5 students during 30-minute sessions once or twice monthly for 10 months on a group project based on any aspect of health care, medical education, or health care improvement. Project outcomes encompassed collaborative learning, teamwork, and communication skills, as well as the process of academic scholarship. During the last week of October 2025, students gave oral and poster presentations of these projects and submitted final written project reports. Projects were evaluated and graded based on standardized criteria provided by UQ, a process I was personally privileged to participate in.

Overall, each team presented outstanding and innovative projects during a 3-day period. I was impressed by what they were able to accomplish in a short time frame. Data from the Epic electronic medical record were used in many of these projects, leading to impressive sample sizes in many of these studies.

I wish to congratulate the students on the conclusion of these projects, as well as staff in the Ochsner Academics/Research Division for their work on these projects that included access to bioinformatics and statistical analyses. Finally, I wish to thank the faculty supervisors for supporting and inspiring the next generation of UQ-OCS clinicians while contributing to innovative medical and health improvement projects in their own fields of expertise.

The strong Ochsner faculty supervisor support will lead to real-world impact. This special section of *Ochsner Journal* includes a sample of the final abstracts submitted by the teams for your academic reading pleasure.

Ronald Amedee, MD, FACS

Director, The University of Queensland-Ochsner Clinical School

Editor-in-Chief, *Ochsner Journal*

## The Impact of Sarcopenia on Postoperative Outcomes in Obese Liver Transplant Recipients

### Sungkyung Linda Kim, BS,^1^ Priyanka Susarla, BS,^1^ Billie Bachewicz, MS,^1^ Alexandra Baldassaro, MS,^1^ Karren Kalai, BA,^1^ Dennis Sonnier, MD^2^

#### ^1^The University of Queensland Medical School, Ochsner Clinical School, New Orleans, LA ^2^Multi-Organ Transplant Institute, Ochsner Clinic Foundation, New Orleans, LA

**Objective:** The purpose was to investigate the impact of sarcopenia on postoperative outcomes in obese adults undergoing liver transplantation.

**Background:** In 2024, over 11,000 liver transplants were performed in just the United States alone. However, there are still over 9,000 individuals waiting for a liver transplant. This illustrates how necessary this procedure is; it is often the only curative treatment for common liver conditions. The mismatch between organ supply and demand highlights the importance of optimizing outcomes for every transplant performed by identifying specific subpopulations at risk for poor posttransplant outcomes. Two such subpopulations are individuals with sarcopenia and those with obesity. Both conditions are associated with an increased incidence of complications related to liver transplant. Preliminary studies suggest that sarcopenic obesity is associated with increased mortality after liver transplantation, potentially exceeding the risk caused by obesity alone. However, evidence remains limited, leaving a significant gap in the literature. This study aims to assess the independent impact of sarcopenia on postoperative outcomes in obese liver transplant recipients.

**Methods:** This retrospective cohort study examined postoperative outcomes in obese adult patients with and without sarcopenia undergoing liver transplantation at Ochsner Health in New Orleans, Louisiana, between 2014 through May 2025. Sarcopenia was defined by reduced skeletal muscle mass and quantified using the skeletal mass index (SMI), calculated as the total cross-sectional muscle area at the L3 vertebral level divided by the patient's height squared (cm^2^/m^2^) on pretransplant abdomen computed tomography scans. Muscle measurements were obtained using CoreSlicer, an open-access validated imaging analysis software.

**Results:** The cohort included 47 individuals with sarcopenia and 28 individuals without sarcopenia. Hospital and intensive care unit (ICU) length of stay posttransplant analysis indicated no significant differences in hospital length of stay between patients with sarcopenia (20 days) and those without (19 days; *P*=0.785) or in ICU length of stay (9.8 vs 8.2 days; *P*=0.779). One-year patient survival was 85.7% for patients with sarcopenia and 85.1% in those without. Three-year patient survival was 82.1% for patients with sarcopenia and 80.8% for patients without (*P*=0.790).

**Discussion:** The study examined postoperative outcomes and survival in obese liver transplant recipients with and without sarcopenia at a high-volume transplant center. Contrary to our initial hypothesis, no statistically significant differences were observed in hospital length of stay, ICU length of stay, or 1- and 3-year survival probabilities between obese patients with sarcopenia and those without. These findings contribute to the growing body of literature regarding the prognostic value of sarcopenia in liver transplantation, particularly among obese patients.

**Conclusion:** Although this study did not identify statistically significant differences in postoperative outcomes, the clinical implications of sarcopenia remain important. Early identification of sarcopenia allows for targeted prehabilitation strategies. Even in obese patients, improving muscle mass may help mitigate metabolic risk and enhance postoperative recovery. A possible explanation for the lack of difference observed is the selection of liver transplant patients. The data were limited to class III obese individuals (body mass index ≥40 kg/m^2^). In a population characterized by severe obesity, the effect of sarcopenia on outcomes may be overshadowed by the metabolic and hemodynamic stress caused by obesity itself. In this study, sarcopenia was treated as a binary variable using SMI thresholds derived from prior literature, which do not account for degrees in muscle depletion. Stratifying sarcopenia into mild, moderate, and severe categories may provide greater discriminative power. The absence of a clear survival difference in our study should not be interpreted as evidence that sarcopenia lacks clinical relevance. Rather, it highlights the complexity of interplay with obesity and the need for more nuanced risk stratification tools that combine both muscle and fat metrics.

## Drug Trends in New Orleans: A One-Year Retrospective Analysis of Emergency Department Urine Drug Screens

### David Galarneau, MD,^1,2^ Emma Trussell, BA,^2^ Gabrielle Herbst, BS,^2^ Holly Coffey, MS,^2^ John Martone, MS,^2^ Paige Polak, MS^2^

#### ^1^Department of Psychiatry, Ochsner Clinic Foundation, New Orleans, LA ^2^The University of Queensland Medical School, Ochsner Clinical School, New Orleans, LA

**Objective:** This study aimed to characterize urine drug screen (UDS) results across emergency departments (EDs) within a tertiary hospital system in New Orleans, Louisiana, to inform clinical decision-making and public health strategies.

**Background:** Substance misuse is a critical public health challenge in New Orleans, with disproportionately high rates of drug-related morbidity and mortality. Opioids, stimulants, and cannabis frequently contribute to ED visits. UDS results in EDs provide real-time insight into local substance use patterns, yet limited research has characterized these trends in New Orleans. Understanding local patterns is essential to guide clinical care, prioritize public health interventions, and optimize testing protocols.

**Methods:** A retrospective cohort study was conducted, including adult ED patients (>18 years) with UDS results from March 2024 to March 2025, across 4 EDs within a tertiary hospital system. The UDS panel was expanded beyond the standard 5-panel screen to include amphetamines, tetrahydrocannabinol (THC)/cannabinoids, cocaine, opiates (including synthetic opioids and fentanyl), phencyclidine (PCP), benzodiazepines, and barbiturates. Deidentified data were extracted from electronic medical records using SlicerDicer. Frequencies of positive results and total tests ordered were calculated for each substance.

**Results:** Of 127,925 adult ED presentations, 13,023 (10.2%) had a UDS ordered, with 6,887 (52.9%) positive. THC (n=3,872, 40.9%) and opioids (n=1,831, 19.3%) were the most frequently detected, followed by stimulants (cocaine n=1,534, 16.2%; amphetamines n=1,418, 15.0%). Fentanyl (n=275, 2.9%) and PCP (n=1, 0.01%) were detected less frequently.

**Discussion:** The predominance of THC, opioids, and stimulants among positive UDS results highlights these substances as the primary drivers of substance exposure in ED patients in New Orleans. The relatively low detection of fentanyl likely reflects selective ordering and limitations of standard UDS panels rather than true absence of use. These findings underscore the importance of aligning ED testing practices with local drug trends and reinforce the role of UDS results in supporting both clinical decision-making and real-time epidemiologic surveillance.

**Conclusion:** THC, opioids, and stimulants are high-priority targets for ED clinical awareness and public health interventions in New Orleans. Fentanyl testing is underordered, highlighting the need for revised protocols or physician training. The high positivity rate underscores the UDS as a valuable tool for clinical decision-making and epidemiologic monitoring.

## Designing a Hearing Impairment Learning Module for Health Care Providers

### Sanil P. Reddy, MS,^1^ Zara Mayat, MS,^1^ Hsuan-Yu Chen, BS,^1^ Jaeho Keum, BS,^1^ Emory Swanger, BS,^1^ Michelle Puzdrakiewicz, MD^1,2^

#### ^1^The University of Queensland Medical School, Ochsner Clinical School, New Orleans, LA ^2^Department of Hospital Medicine, Ochsner Clinic Foundation, New Orleans, LA

**Objective:** With guidance from community organizations and subject matter experts, we aimed to create an educational module detailing the etiology, diagnosis, audiologic evaluation, and management of hearing impairment.

**Background:** Hearing impairment is projected to impact 2.45 billion people globally by 2050. Standardized screening of hearing impairment is crucial to diagnose and prevent poor developmental, psychiatric, and socioeconomic outcomes. However, health care providers do not often receive structured training on its identification and burden. An educational module can optimize management of hearing impairment by liaising community and expert guidance on its screening, diagnosis, and management.

**Methods:** A narrative literature review and 5 interviews with subject matter experts in Orleans Parish and Jefferson Parish in Louisiana were conducted between April and August 2025. Information shared by 4 medical specialists was used to refine our literature review. A service leader at the Louisiana Association of the Deaf and St. Gerard Community for the Deaf provided resources and community engagement opportunities catered to the hearing-impaired community. With their guidance, a slide deck was created, serving as a blueprint for a future interactive learning module.

**Results:** A collective understanding from literature review and interviews of subject matter experts revealed that the current orthodoxy in addressing hearing impairment during medical education strongly relies on anatomy, physiology, and management guidelines. An educational module with a holistic approach, entailing opportunities for early detection, multidisciplinary care, and community resources, can optimize patient care.

**Discussion:** Initiatives to diagnose and treat hearing impairment begin during medical education and extend to practicing health care providers. The interactive module is designed to summarize the pathophysiology of hearing impairment, practice recommendations, and community engagement opportunities. The preliminary slide deck increases access to health care providers and provides opportunity for feedback. As the module is piloted, perspectives from patients and caregivers will also be incorporated.

**Conclusion:** Community-specific educational modules for health care providers can ameliorate the underrecognized, impending burden of hearing impairment.

## Automated Diagnosis Alerts to Enhance Problem List Completeness and Comorbid Conditions Rate Capture

### Ruchee Shrestha, MPH,^1^ Antonio Del Vecchio, BS,^1^ Ashwin Kalyanakumar, BS,^1^ Rahim Abdul, MS,^1^ Shirley Chen, BS,^1^ Jason Hill, MD, MMM,^1,2^ Haroon Jakher, MD^1,2^

#### ^1^The University of Queensland Medical School, Ochsner Clinical School, New Orleans, LA ^2^Department of Hospital Medicine, Ochsner Clinic Foundation, New Orleans, LA

**Objective:** This study compared the frequency of automated electronic health record (EHR) diagnostic alerts and provider acceptances between an academic and community hospital setting.

**Background:** Accurate problem lists enhance patient safety, cost efficiency, and diagnostic guidance. EHR interventions can improve problem list completeness, but differences between academic and community settings remain poorly characterized.

**Methods:** We conducted a retrospective quality improvement study of 11 EHR diagnostic alerts for adult patients from February to July 2025, at 1 academic and 1 community hospital. Primary outcomes included alert frequency and problem list incorporation. Hospital and diagnosis differences were compared, respectively, using 2-sample proportions tests and Pearson chi-square analyses. Logistic regression assessed independent effects of hospital and diagnosis on alert acceptance.

**Results:** There were 20,577 total alerts, and 9.87% were accepted (Figure). Chi-square analyses showed the largest significant between-hospital differences for anemia (χ^2^=29.7, *P*<0.001) and hyponatremia (χ^2^=24.5, *P*<0.001). Logistic regression showed significantly lower alert acceptance at the community hospital (odds ratio=0.575, *P*<0.001). Hypokalemia, thrombocytopenia, hypomagnesemia, hypophosphatemia, and obesity had odds ratios of 1.23 to 1.41 (*P*≤0.041) of acceptance compared to anemia.

**Figure.**
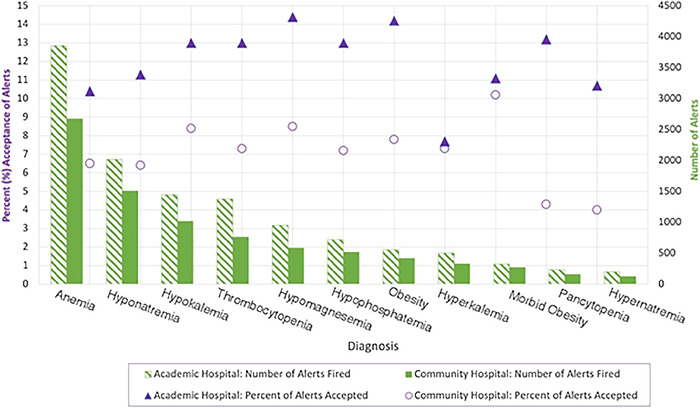
Number of automated alerts fired and proportion accepted for each diagnosis by hospital setting.

**Discussion:** Hospital setting and diagnosis independently influenced alert acceptances. Persistent differences suggest context affects engagement. Similar hyperkalemia and morbid obesity acceptance rates likely reflect regional prevalence.

**Conclusion:** There were 42.5% lower odds of automated diagnostic alert acceptance at the community hospital compared to the academic hospital. Hospital setting and diagnosis independently predicted alert acceptance.

## Evaluating the Effect of Enrollment in a Home Digital Blood Pressure Monitoring Program on 30-Day Readmission Following Hospitalization

### Bhargav Venkatraghavan, BS,^1^ Ajay Tupil, BS,^1^ Daniel Beidokhti, BS,^1^ Dhiren Suryadevara, BS,^1^ Rahul Hadap, MS,^1^ Sahil Bharwani, DO, MPH,^2^ Kristen R. Toups, MD, MPH, FACP, FHM^1,3^

#### ^1^The University of Queensland Medical School, Ochsner Clinical School, New Orleans, LA ^2^Department of Internal Medicine, Ochsner Clinic Foundation, New Orleans, LA ^3^Department of Hospital Medicine, Ochsner Clinic Foundation, New Orleans, LA

**Objective:** This study evaluated the association between enrollment in a home-based digital hypertension management program among hospitalized patients at the time of hospital discharge and subsequent 30-day all-cause readmissions.

**Background:** Hospital readmissions within 30 days of discharge are a key quality metric and a target for improvement initiatives. Our academic medical center all-cause 30-day readmission rate for 2024 was 15.35% compared to the national average of 13.9%. Hypertension is a leading risk factor for cardiovascular diseases and is implicated as a primary or contributing cause of deaths in the United States. Prior research shows a high incidence of readmission (19%) for patients with hypertensive disorders. Our health system digital hypertension program, a home-based blood pressure monitoring program supported by a primary care provider and pharmacy team, has shown promise in reducing all-cause hospitalizations and emergency department visits for ambulatory patients, but its impact when initiated at hospital discharge remains unexplored. We embarked on a pilot program to expand enrollment in the digital hypertension program to hospitalized patients.

**Methods:** This was a retrospective chart review of adult patients admitted to our academic medical center between April 2025 and August 2025 with a diagnosis of hypertension who were eligible for enrollment in the digital hypertension program at the time of discharge. Patients were categorized into 2 groups: those referred and enrolled in our digital hypertension program at discharge (enrolled group) and those referred but not enrolled (nonenrolled group). Demographic, clinical, and utilization data were extracted from the electronic medical record. The primary outcome was 30-day all-cause readmissions. Secondary outcomes included 30-day all-cause emergency department visits and primary care follow-up within 30 days of discharge.

**Results:** A total of 491 patients were flagged as eligible by the electronic medical record, of whom 62 met inclusion criteria. Of those, 16 patients (25.8%) enrolled at the time of hospital discharge. The overall 30-day readmission rate was 19.4%. No patients in the enrolled group were readmitted within 30 days, compared with 12 patients (26.1%) in the nonenrolled group (Fisher exact test, *P*=0.024). Primary care follow-up occurred more frequently among enrolled patients (62% vs 26%, *P*=0.015). Emergency department visits were higher among nonenrolled patients (26% vs 25%), although this difference was not statistically significant (*P*=1.00).

**Discussion:** This 5-month pilot program identified that enrollment in our digital hypertension program at the time of discharge may be associated with reduced 30-day all-cause readmissions among patients with hypertension. On bivariable analysis, enrollment in our digital hypertension program was associated with significantly lower 30-day readmissions (0% vs 26%, *P*=0.024). However, primary care follow-up was more frequent among enrolled patients (62% vs 26%, *P*=0.015) and may partially explain the observed difference in readmissions. When adjusted for primary care follow-up in logistic regression, the association between the digital hypertension program enrollment and readmission remained directionally protective, although statistical significance was limited by the small sample size, and regression modeling was limited by zero events in enrollees.

**Conclusion:** Enrolling patients in a digital hypertension program at the time of hospital discharge has identified a promising strategy to reduce hospital readmissions. The positive association observed in this study warrants further evaluation in studies with larger sample sizes to properly estimate the true statistical significance of patient enrollment in the digital hypertension program.

## Educational Modalities and Kidney Graft Survival: An Ecological Study

### Cole Astin, MPH,^1^ Mason Williams, BA,^1^ Turner Simmers, BS,^1^ Bernard Regidor, BS,^1^ Adam Nagourney, BS,^1^ Dennis Sonnier, MD^2^

#### ^1^The University of Queensland Medical School, Ochsner Clinical School, New Orleans, LA ^2^Multi-Organ Transplant Institute, Ochsner Clinic Foundation, New Orleans, LA

**Objective:** This study aimed to determine the efficacy of different pretransplant educational delivery methods on kidney graft survival at 1 and 3 years.

**Background:** Existing evidence suggests that pretransplant patient education leads to improved kidney transplant outcomes. A previous study demonstrated that increased pretransplant education leads to greater immunosuppressive compliance. Additional research also determined that greater education and psychosocial support lead to a return to normalcy for kidney transplant patients. Despite these contributions, only a small number of studies have investigated the efficacy of specific educational delivery methods.

**Methods:** This study utilized an ecological model to examine 1- and 3-year graft survival in adult kidney transplant recipients using data from the Scientific Registry of Transplant Recipients. Adult transplant recipients from US-based transplant centers completing at least 100 adult kidney transplants were included. Qualitative data concerning the delivery style of patient education were sourced from the transplant centers’ websites. Educational delivery methods, including in-person, video-based, print, or multimodal, were identified through institutional websites or via phone calls and were categorized using a logistics regression model. The principal outcome was 1- and 3-year graft survival rates. Quantitative data were analyzed using the STATA software.

**Results:** This study hypothesized that all educational delivery methods were associated with improved graft survival at 1 and 3 years posttransplant compared to the standard of care. Centers employing multimodal education strategies were expected to demonstrate statistically significant differences from other modalities. After statistical analysis, we found that there was not a statistically significant difference between the standard of care and additional education or between any education modality.

**Discussion:** The ecological analysis of 147 US kidney transplant centers revealed no statistically significant differences in 1- or 3-year graft survival between centers utilizing the standard of care educational approach and those incorporating additional modalities such as printed materials, in-person education, video only, combined online and in-person courses, and a multimodal approach. These findings suggest that the standard educational pathway provided at transplant centers is comprehensive, sufficient, and can produce adequate early graft survival outcomes. Furthermore, additional statistical analysis demonstrated no significant changes in survival outcomes from the 1- to 3-year period when comparing each educational modality. Despite this, education can still improve patient understanding, satisfaction, and preparedness even if survival is unchanged. Collectively, the results indicate that while supplemental educational interventions are safe and well-intentioned, they may not yield measurable improvements in graft longevity at an institutional level.

**Conclusion:** This study found no meaningful difference in 1- or 3-year graft survival between the standard education pathway and centers that added additional education (videos, print, classes, mixed formats, or a multimodal approach), suggesting a mature, multidisciplinary standard of care is sufficient to deliver positive early outcomes. Overall, reliably delivering a comprehensive standard of care likely matters more for early graft survival than any single format, but intelligent, tailored education can still improve patient understanding, experience, and long-term success.

